# Expression Profile of New Marker Genes Involved in Differentiation of Human Wharton’s Jelly-Derived Mesenchymal Stem Cells into Chondrocytes, Osteoblasts, Adipocytes and Neural-like Cells

**DOI:** 10.3390/ijms241612939

**Published:** 2023-08-18

**Authors:** Katarzyna Stefańska, Lucie Nemcova, Małgorzata Blatkiewicz, Agnieszka Żok, Mariusz Kaczmarek, Wojciech Pieńkowski, Paul Mozdziak, Hanna Piotrowska-Kempisty, Bartosz Kempisty

**Affiliations:** 1 Department of Histology and Embryology, Poznan University of Medical Sciences, 60-781 Poznan, Poland; 2 Cellivia 3 S.A., 61-623 Poznan, Poland; 3 Institute of Animal Physiology and Genetics of the Czech Academy of Sciences, 27721 Libechov, Czech Republic; 4 Division of Philosophy of Medicine and Bioethics, Poznan University of Medical Sciences, 60-806 Poznan, Poland; 5 Department of Cancer Immunology, Poznan University of Medical Sciences, 61-866 Poznan, Poland; 6 Gene Therapy Laboratory, Department of Cancer Diagnostics and Immunology, Greater Poland Cancer Centre, 61-866 Poznan, Poland; 7 Division of Perinatology and Women’s Diseases, Poznan University of Medical Sciences, 60-535 Poznan, Poland; 8 Prestage Department of Poultry Sciences, North Carolina State University, Raleigh, NC 27695, USA; 9 Department of Toxicology, Poznan University of Medical Sciences, 60-631 Poznan, Poland; 10 Department of Basic and Preclinical Sciences, Institute of Veterinary Medicine, Nicolaus Copernicus University in Torun, 87-100 Torun, Poland; 11 Department of Veterinary Surgery, Institute of Veterinary Medicine, Nicolaus Copernicus University in Torun, 87-100 Torun, Poland; 12 Division of Anatomy, Department of Human Morphology and Embryology, Wroclaw Medical University, 50-368 Wroclaw, Poland; 13 Department of Obstetrics and Gynecology, University Hospital and Masaryk University, 60177 Brno, Czech Republic; 14 Physiology Graduate Faculty, North Carolina State University, Raleigh, NC 27695, USA

**Keywords:** Wharton’s jelly, mesenchymal stem cells, RNA-seq, MSC, differentiation

## Abstract

Wharton’s jelly (WJ) contains mesenchymal stem cells (MSCs) exhibiting broad immunomodulatory properties and differentiation capacity, which makes them a promising tool for cellular therapies. Although the osteogenic, chondrogenic and adipogenic differentiation is a gold standard for proper identification of MSCs, it is important to elucidate the exact molecular mechanisms governing these processes to develop safe and efficient cellular therapies. Umbilical cords were collected from healthy, full-term deliveries, for subsequent MSCs (WJ-MSCs) isolation. WJ-MSCs were cultivated in vitro for osteogenic, chondrogenic, adipogenic and neurogenic differentiation. The RNA samples were isolated and the transcript levels were evaluated using NovaSeq platform, which led to the identification of differentially expressed genes. Expression of *H19* and *SLPI* was enhanced in adipocytes, chondrocytes and osteoblasts, and *NPPB* was decreased in all analyzed groups compared to the control. *KISS1* was down-regulated in adipocytes, chondrocytes, and neural-like cells compared to the control. The most of identified genes were already implicated in differentiation of MSCs; however, some genes (*PROK1*, *OCA2*) have not yet been associated with initiating final cell fate. The current results indicate that both osteo- and adipo-induced WJ-MSCs share many similarities regarding the most overexpressed genes, while the neuro-induced WJ-MSCs are quite distinctive from the other three groups. Overall, this study provides an insight into the transcriptomic changes occurring during the differentiation of WJ-MSCs and enables the identification of novel markers involved in this process, which may serve as a reference for further research exploring the role of these genes in physiology of WJ-MSCs and in regenerative medicine.

## 1. Introduction

The human umbilical cord fulfills a crucial role in maintaining pregnancy, providing the blood supply for the fetus and participating in biological waste removal [1]. The three umbilical vessels, namely, two arteries and one vein, are critically important. Interestingly, the umbilical vein contains mesenchymal stem cells (MSCs) fulfilling the criteria established by International Society for Cellular Therapy (ISCT), meaning they are plastic-adherent, possess capability to differentiate into adipocytes, chondrocytes and osteoblasts and express specific markers, such as CD90, CD105 (endoglin) and CD73, while not expressing CD45, CD34, CD14 or CD11b, CD79α or CD19 and HLA-DR surface molecules [2]. The confirmation of differentiation may require specific staining or RT-PCR to detect the presence of aggrecan in chondro-induced MSCs, lipid droplets in adipo-induced MSCs, calcium deposits or osteocalcin in osteo-induced MSCs and Nissl bodies in neuro-induced MSCs [2,3].

The umbilical vessels lack tunica adventitia, which is replaced by Wharton’s jelly, a mucoid connective tissue preventing the vessels from torsion [4]. Fibroblastoid cells located in Wharton’s jelly also fulfill the minimal criteria for MSCs and provide an even better source of cells that may be utilized in cellular therapies. Wharton’s jelly-derived MSCs (WJ-MSCs) are the richest in stem cell properties amongst cells obtained from the other parts of umbilical cord; moreover, WJ-MSCs are highly proliferative [5]. Beyond the aforementioned markers, WJ-MSCs express CD29, CD44, CD146 and early embryonic transcription factors (Nanog, Oct-4, Sox-2) [6,7]. In addition, WJ-MSCs exhibit broad immunomodulatory properties, and the potential of these cells to differentiate into osteogenic and chondrogenic lineages is greater than of bone marrow-derived MSCs (BM-MSCs) [8,9]. Recently, da Rosa et al. [10] reported the pulmonary differentiation of WJ-MSCs in the form of spheroids for drug testing.

The unique properties of WJ-MSCs create a possibility to utilize these cells in regenerative medicine. It seems that especially the field of engineered scaffolds seeded with WJ-MSCs or 3D-printed tissue constructs is rapidly evolving in recent years. Zhang et al. [11] reported the construction of artificial periosteum with methacrylamide gelatin hydrogel and Wharton’s jelly microparticles for bone regeneration, which resulted in BM-MSCs recruitment and promotion of cell proliferation in rats. Mansour et al. [12] utilized the fetal bovine acellular dermal matrix seeded with WJ-MSCs for healing full-thickness skin wounds in rats. Ohers researchers demonstrated the electrospun poly (ε-caprolactone) and collagen scaffolds seeded with WJ-MSCs for skin tissue regeneration [13], silk and gelatin nanofibrous 3D scaffolds for differentiation of WJ-MSCs into islet-like cells [14], and many others. Given the vast possibility to use WJ-MSCs in regenerative medicine, the constant increase in the number of clinical trials utilizing these cells is not surprising. In addition, MSCs derived from this source fail to create ethical controversies, as in the case of embryonic stem cells (ESCs), whose acquisition requires embryo destruction [15]. Although the tri-lineage differentiation of WJ-MSCs has been performed multiple times [5,16,17,18], it is important to elucidate the molecular mechanisms governing the plasticity and differentiation of these cells, especially during an in vitro culture, since cells utilized in clinical environment often must be propagated in vitro prior to the transplantation. Furthermore, the possibility to construct 3D scaffolds seeded with WJ-MSCs, which potentially might be used in clinical environment, also requires in-depth understanding of the molecular events occurring in cultured cells. RNA sequencing (RNA-seq) enables the analysis of transcriptomic changes occurring during an in vitro differentiation of WJ-MSCs. Hence, this study focuses on the most differentially expressed genes in osteo-, chondro-, adipo- and neuro-induced WJ-MSCs as compared to undifferentiated cells, to identify new markers associated with these processes.

## 2. Results

### 2.1. Morphological Analysis

**Figure 1:**
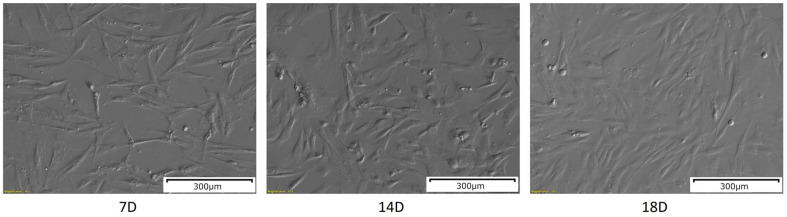
The results of the morphological analysis of the WJ-MSCs primary culture at 7, 14 and 18 days of in vitro culture. The pictures were taken at a 10× magnification. Scale bar: 300 µm.

The WJ-MSCs after 7 days of in vitro culture were plastic-adherent and presented an elongated, fibroblast-like shape (Figure 1), which is characteristic for MSCs. After 14 days of in vitro culture, the number of cells was visibly increased, as well as their size. After another four days of culture, on day 18, the cells exhibited more flattened morphology, and the confluency was increased.

### 2.2. Evaluation of WJ-MSCs Differentiation

After the third passage, the WJ-MSCs were subjected to osteogenic, chondrogenic, adipogenic and neurogenic differentiation. The results of the differentiation were confirmed with specific staining and presented in previous articles [19]. Briefly, in adipo-induced WJ-MSCs stained with Oil Red O, the red lipid droplets were observed, which were not present in the control sample. Neuro-induced WJ-MSCs were stained with cresyl violet, which revealed the presence of Nissl bodies in contrast to the control sample. Osteo-induced WJ-MSCs after Alizarin Red staining exhibited the presence of red-colored calcium deposits, while Alcian Blue staining revealed the presence of intensely blue-colored cartilage extracellular matrix in chondro-induced spheroids. In conclusion, the results indicate that the four-lineage differentiation of WJ-MSCs was successful.

### 2.3. Flow Cytometry Analysis

**Figure 2:**
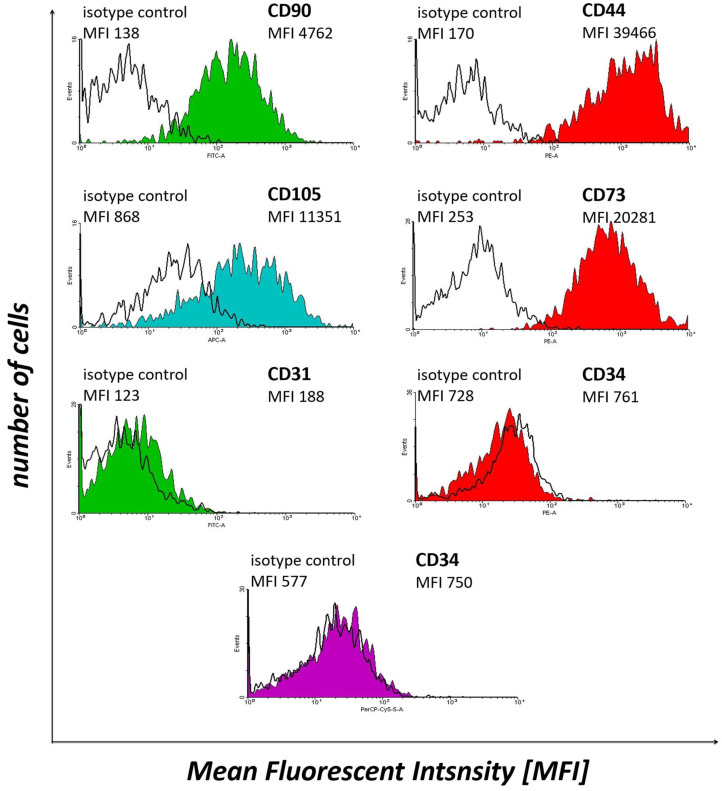
The results of flow cytometry analysis of selected WJ-MSCs markers, i.e., CD90, CD44, CD105, CD73, CD31 and CD34, in the cell samples subjected to in vitro culture.

WJ-MSCs expressed CD90 (Thy-1), CD44, CD105 (endoglin) and CD73 (5′-nucleotidase), while not expressing CD31 (platelet endothelial cell adhesion molecule) and CD34 (Figure 2). These results confirm the MSC-like character of Wharton’s jelly-derived cells. Therefore, these samples were subjected to further experiments.

### 2.4. RNA-seq Analysis

**Figure 3:**
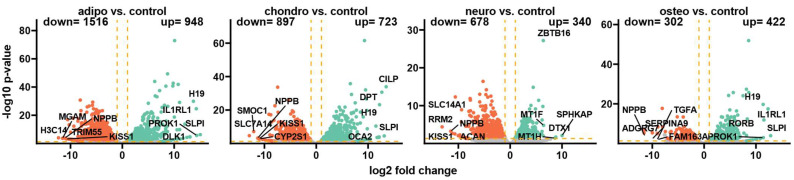
The volcano plots display the expression patterns of different experimental groups. Each point represents the average expression level of a particular gene. The orange dotted lines indicate the established cut-off values based on the parameters: |fold change| = 2 and *p*-value = 0.05. Genes located above these cut-off lines are considered differentially expressed and are represented as red (down-regulated) and green (up-regulated) dots. The numbers of up-regulated and down-regulated genes are provided in the top right and top left corners, respectively. Additionally, the five most significantly differentially expressed genes are highlighted in the plot.

Following the differentiation process and RNA sequencing, Bioconductor’s online packages were applied to examine the overall changes in the transcriptome. Initially, the general expression profile of these transcriptome changes was assessed and visualized as volcano plots (Figure 3). By applying predefined criteria for identifying differentially expressed genes (|fold change| = 2 and *p*-value < 0.05), a total of 948 up-regulated genes (representing the highest number of overexpressed genes) and 1516 down-regulated genes in adipocytes compared to the control group were identified. Moreover, the comparison of chondrocytes to control revealed that 723 genes were up-regulated and 897 genes were down-regulated, while in neural-like cells vs. control, there were 340 up-regulated and 678 down-regulated genes. The comparison of osteoblasts to control indicates 422 up-regulated genes and 302 down-regulated genes, which was the lowest number across the whole analysis.

For the group of adipocytes, the study revealed that the top five up-regulated genes included *H19*, *IL1RL1*, *PROK1*, *SLPI* and *DLK1*, while the most down-regulated genes were *MGAM*, *NPPB*, *H3C14*, *TRIM55* and *KISS1*. In the group of chondrocytes, there was an up-regulation of *CILP*, *DPT*, *H19*, *SLPI* and *OCA2* with a down-regulation of *NPPB*, *SMOC1*, *SLC7A14*, *KISS1* and *CYP2S1*. The comparison of neural-like cells to control revealed overexpression of *ZBTB16*, *MT1F*, *MT1H*, *DTX1* and *SPHKAP*, while the expression of *SLC14A1*, *RRM2*, *NPPB*, *KISS1* and *ACAN* was down-regulated. Furthermore, in the comparison of osteoblasts and control, *H19*, *IL1RL1*, *SLPI1* and *PROK1* were overexpressed, with suppressed expression of *NPPB*, *TGF1*, *SERPINA9*, *ADGRG7* and *FAM163*.

In summary, several genes were expressed commonly across all analyzed groups. Expression of *H19* and *SLPI* was enhanced in adipocytes, chondrocytes and osteoblasts, and *NPPB* was decreased in all analyzed groups compared to the control. *KISS1* was down-regulated in adipocytes, chondrocytes and neural-like cells compared to control.

**Figure 4:**
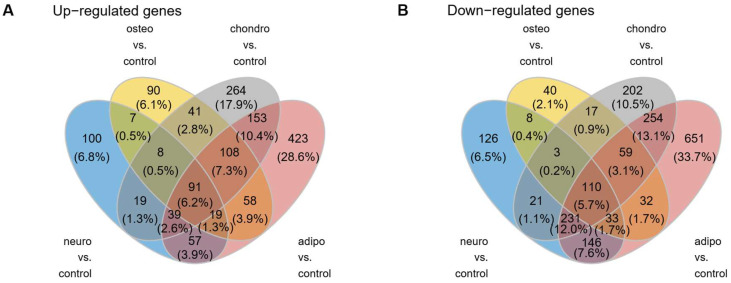
The Venn diagrams illustrate the overlap and unique genes that undergo up- (**A**) and down-regulation (**B**) across all analyzed groups compared to controls.

As can be observed in the Venn diagram, some genes overlap between the compared experimental conditions (Figure 4). For all analyzed groups, 91 genes (6.2%) were up-regulated and 110 (5.7%) were down-regulated, compared to the control group.

**Figure 5:**
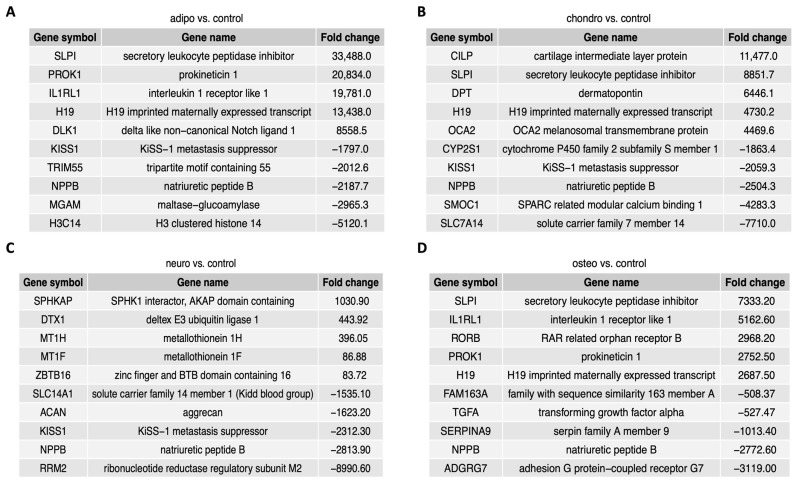
The figure presents a list of ten genes, with five genes displaying the highest expression levels and five genes exhibiting the lowest expression levels, comparing the adipocytes (**A**), chondrocytes (**B**), neural-like cells (**C**) and osteoblasts (**D**) to the control.

Figure 5 contains a list of the top ten genes with the highest (five genes) and lowest (five genes) expression fold change in adipocytes, chondrocytes, neural-like cells and osteoblasts in contrast to controls as well as a comparison between the groups. The fold change values of the top five overexpressed genes in the comparison of adipocytes to control (Figure 5A) ranged from 8558.5 to 33,488.0, while the expression of the down-regulated genes ranged from −5120.1 to −1797.0. In chondrocytes vs. control (Figure 5B), the fold change values of the top five up-regulated genes ranged from 4469.6 to 11,477.0, while the expression of top five down-regulated genes ranged from −7710.0 to −1863.4. The comparison of neural-like cells to control (Figure 5C) revealed the top five up-regulated genes with fold change values ranging from 1030.9 to 86.88, and top ten down-regulated genes, with fold change values ranging from −8990.60 to −1535.10. In osteoblasts vs. control (Figure 5D), the fold change for up-regulated genes ranged from 7333.20 to 2687.50, while for down-regulated genes, the fold change ranged from −3119.0 to −508.37. Commonly down-regulated gene in all differentiated cells was *NPPB*, while *SLPI* and *H19* were up-regulated in adipo-, chondro- and osteo-induced WJ-MSCs.

**Figure 6:**
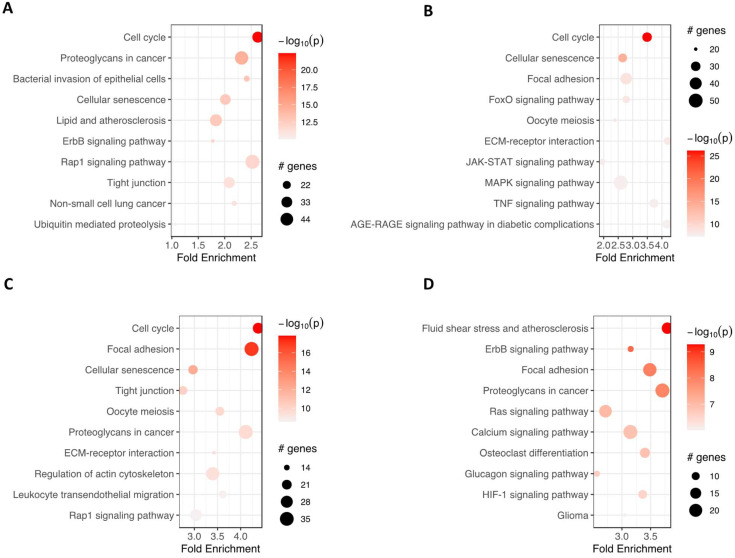
The bubble chart illustrates the enrichment results for four groups: adipocytes (**A**), chondrocytes (**B**), neural-like cells (**C**) and osteoblasts (**D**), compared to the control group. The *x*-axis represents the fold enrichment values, while the *y*-axis represents the enriched terms. The size of each bubble indicates the number of significant genes associated with the specific enriched term. The color of the bubbles corresponds to the −log10 (lowest-*p*) value, with shades closer to red indicating a higher level of significance in the enrichment. Abbreviations: # genes—number of genes.

Furthermore, pathfindR was used to investigate which enrichment term was mainly regulated in analyzed groups of cells (Figure 6). ‘Cell cycle’ and ‘cellular senescence’ were amongst the most significantly enriched terms in adipocytes, chondrocytes and neural-like cells compared to controls. ‘Proteoglycans in cancer’ were enriched commonly in the group of adipocytes, neural-like cells and osteoblasts vs. controls. At the same time, ‘focal adhesion’ was improved in the group of chondrocytes, neural-like cells and osteoblasts.

**Figure 7:**
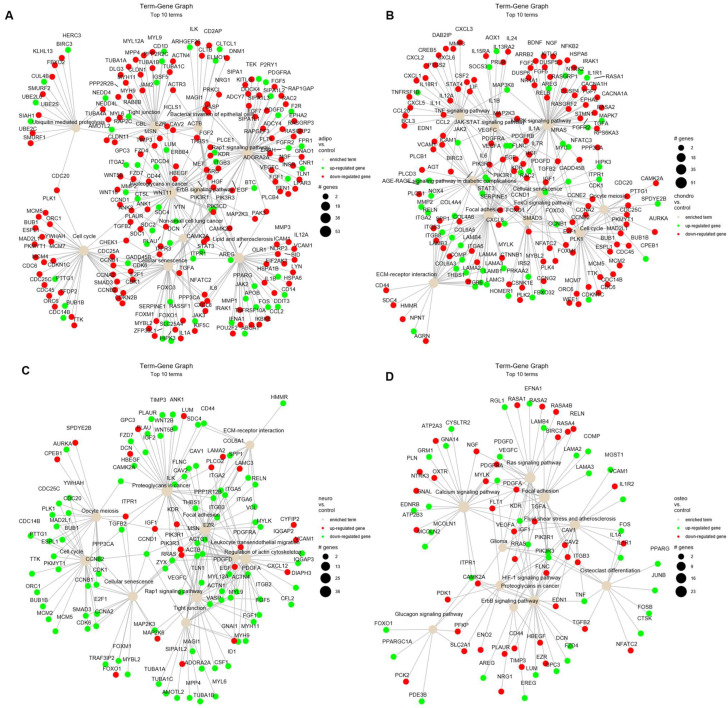
The term–gene graph for significant genes involved in the enriched terms for adipocytes (**A**), chondrocytes (**B**), neural-like cells (**C**) and osteoblasts (**D**) compared to control. The node sizes are plotted proportional to the number of genes a term contains, where the node color indicates up-regulated (green) and down-regulated genes (red).

Moreover, the relations of terms with significant genes were investigated to better understand the intricate relationships and regulatory networks operating within the biological processes identified in previous analysis (Figure 7). The analysis of adipocytes vs. control shows an enrichment of many terms, such as ‘cell cycle’, ‘cellular senescence’ and ‘tight junctions’ (Figure 7A). Similar for the analysis of chondrocytes vs. control, we indicate down-regulation of genes related to ‘cell cycle’ and ‘oocyte meiosis’, as well as ‘TNF’ and ‘MAPK’ signaling pathway (Figure 7B). The comparison of neural-like cells to controls revealed activation of ‘cell cycle’, ‘oocyte meiosis’, ‘cellular senescence’ and ‘tight junctions’ (Figure 7C). In the comparison of osteoblasts vs. control, the mostly activated term was ‘osteoclast differentiation’ (Figure 7D). The highest number of up-regulated genes have been identified on the term–gene graph for neural-like vs. controls (Figure 7C), while the mostly down-regulated genes were in adipocytes compared to controls (Figure 7A).

**Figure 8:**
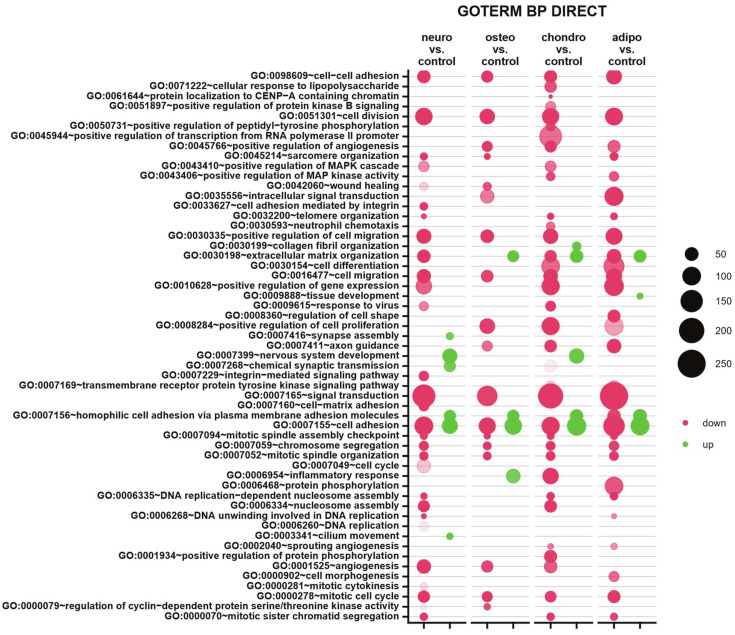
Compared to the control, the bubble plot of overrepresented biological processes pathway enrichment analyses in the DAVID GO BP DIRECT annotations database represents the enriched functions of gene expression profiles between adipocytes, chondrocytes, neural-like and osteoblasts. The size of each bubble corresponds to the number of differentially expressed genes associated with the respective GO BP terms. The transparency of each bubble corresponds to the *p*-value, with greater transparency indicating proximity to the *p* = 0.05 cut-off value. Green bubbles represent overexpressed genes, while red bubbles depict down-regulated genes. The graph shows only the GO groups above the establisher cut-off criteria (*p* with correction < 0.05, a minimal number of genes per group > 2).

Subsequently, to provide an insight into differentially expressed genes in the specific biological processes, the up- and down-regulated genes were analyzed from all studied groups and assigned to Gene Ontology (GO) terms for classification based on biological processes (GO term BP) (Figure 8). The comparison of neural-like cells to control revealed 29 down-regulated and 6 up-regulated processes. In neural-like cells, the highest statistical significance was revealed for ‘cell division’ (GO:0051301, *p* with Benjamini correction = 6.83 × 10^−14^), while the most activated was ‘homophilic cell adhesion via plasma membrane adhesion molecules’ (GO:0007156, *p* with Benjamini correction = 1.03 × 10^−6^). For the osteoblasts vs. control comparison, from all 18 processes, mostly down-regulated processes were ’cell division’ (GO:0051301, *p* with Benjamini correction = 1.46 × 10^−9^) and ‘cell adhesion’ (GO:0007155, *p* with Benjamini correction = 1.9 × 10^−9^). Moreover, mostly up-regulated process (from four processes) in the group of osteoblasts was ‘cell adhesion’ (GO:0007155, *p* with Benjamini correction = 3.19 × 10^−9^). Also, the analysis of chondrocytes and adipocytes indicates similarities to neural-like cells. In the group of chondrocytes, 35 down- and 5 up-regulated processes were observed, from which the most inhibited was ‘signal transduction’ (GO:0007165, *p* with Benjamini correction = 1.49 × 10^−11^), and most activated ‘cell adhesion’ (GO:0007155, *p* with Benjamini correction = 7.8 × 10^−16^). In the analysis of adipocytes, for both up- and down-regulated processes, the highest significance was assigned to ‘cell adhesion’ process (GO:0007155, *p* with Benjamini correction = 7.8 × 10^−12^ for activated, and *p* with Benjamini correction = 1.9 × 10^−12^ for inhibited). To sum up, some similarities across all analyzed groups were observed. Processes like cell adhesion, cell division, cell migration, positive regulation of cell migration, chromosome segregation, mitotic spindle organization and mitotic cell cycle were down-regulated in all analyzed groups compared to the control. Only two processes were activated in all studied groups, which were homophilic cell adhesion via plasma membrane adhesion molecules and cell adhesion, compared to control.

**Figure 9:**
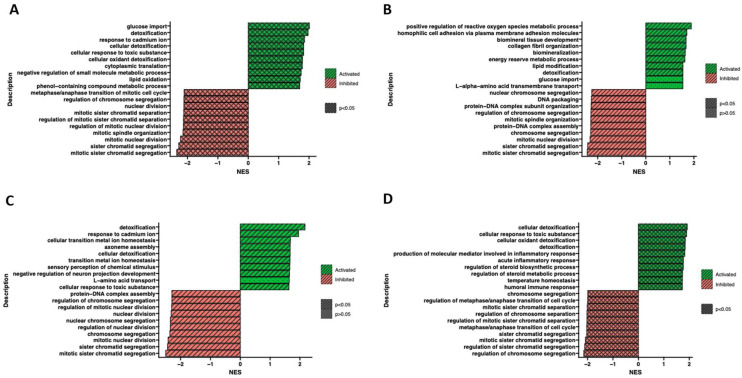
Gene Set Enrichment Analysis (GSEA) of cells in adipocytes (**A**), chondrocytes (**B**), neural-like (**C**) and osteoblast (**D**) cells in comparison to control. Normalized enrichment score (NES) is presented as a bar plot and indicates the enrichment of a gene set at the top of a ranked list (green indicators), and gene sets with a negative NES are overrepresented at the bottom of the gene list (red indicators).

Furthermore, the ontological terms with the highest and the lowest *p*-value were determined by Gene Set Enrichment Analysis (GSEA) to provide information about the functional enrichment of gene sets or predefined gene groups within a dataset (Figure 9). Based on the normalized expression level data, a list of significantly described terms from the Hallmark database software (from the Molecular Signatues Database) (Available at: https://www.cell.com/cell-systems/pdf/S2405-4712(15)00218-5.pdf, accessed on 28 May 2023) was generated. For all analyzed groups, only one activated process (*p* > 0.05) was detoxification. Meanwhile, altered processes related to chromatid and chromosome segregation, regulation of chromosome separation and cell cycle regulation were observed. Despite employing different methodologies, the Gene Set Enrichment Analysis yields results that are relatively consistent and comparable to the findings obtained from analyzing ontological clusters using DAVID. These complementary approaches contribute to a comprehensive understanding of the biological processes and pathways associated with the analyzed gene sets.

**Figure 10:**
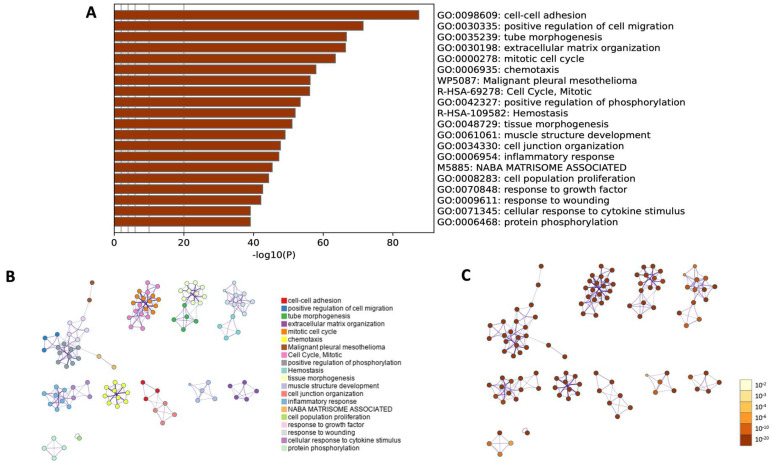
Metascape functional enrichment analysis of differentially expressed genes (DEGs) according to biological processes. (**A**) Bar chart of top 20 clustered enrichment ontology categories (GO and KEGG terms). (**B**) The enrichment ontology clusters, where a circular node depicts each term, while the color of each node represents the cluster identity to which the term belongs. (**C**) The enrichment network, where the nodes are colored based on their *p*-values associated with different shades of color, as illustrated in the accompanying legend. Nodes with darker colors indicate greater statistical significance.

Moreover, a comprehensive functional enrichment analysis was performed using Metascape (Figure 10). The Gene Ontology heatmap provided by Metascape allowed the exploration of the functional enrichment patterns across all differentially expressed genes and provided an insight into biological processes that were overrepresented in the input gene list (Figure 10A). The 20 top statistically enriched GO terms were identified, and the top five enriched processes were cell–cell adhesion (GO:0098609, log10(*p*) = −87.42); positive regulation of cell migration (GO:0030335, log10(*p*) = −71.43); tube morphogenesis (GO:0035239, log10(*p*) = −66.64); extracellular matrix organization (GO:0030198, log10(*p*) = −66.37) and mitotic cell cycle (GO:0000278, log10(*p*) = −63.47).

Next, utilizing an advanced clustering algorithm to group similar functional terms together, the coherent functional clusters within the enrichment results were identified and presented as a network layout (Figure 10B,C). The related biological processes and pathways that may share common underlaying mechanisms were identified. The current findings confirmed the results obtained in previous analyses and showed similar biological processes (GO BP) and their equal statistical significance.

## 3. Discussion

The aim of this study was to identify genes involved in the process of in vitro differentiation of WJ-MSCs towards osteoblasts, chondrocytes, adipocytes and neural-like cells via comparison of the cellular transcriptome utilizing the RNA-seq technique. Both RNA-seq and microarrays techniques were utilized to analyze the eukaryotic transcriptomes in numerous studies [20,21,22], although RNA-seq provides more reproducible results; therefore, this method was chosen in the current study. WJ-MSCs have already been utilized in clinical trials to support the treatment of the spinal cord injury (NCT03003364, NCT04288934), type I diabetes (NCT03973827, NCT03406585), osteoarthritis (NCT03866330, NCT03337243), graft versus host disease (NCT03158896) and many others. However, the cells after transplantation encounter ischemic and nutrient-deprived environment, which may result in cell death [23]. Several researchers suggested that the in vitro differentiation of MSCs might decrease the sensitivity of these cells to apoptosis after the transplantation [24,25], while the others obtained the opposite outcomes [26]. Our previous results obtained after performing RNA-seq on differentiated WJ-MSCs with four lineages indicated that differentiation of WJ-MSCs prior to the transplantation would not be more beneficial than the utilization of undifferentiated cells, considering the expression of apoptosis-related genes in these groups [19]. Conflicting results emphasize the importance of investigation of the molecular processes occurring during the differentiation of MSCs, not only those engaged in the apoptotic process, to decide whether the differentiation conducted prior to the transplantation of cells would be beneficial. A further insight into the transcriptomic changes during in vitro culture and differentiation of WJ-MSCs may contribute to the progress of in vitro tissue models construction for drug testing and is necessary for the development of safe and reliable therapies and predicting their possible outcomes, and will serve as a point of reference for future research.

*SLPI* (secretory leukocyte peptidase inhibitor) was amongst the top five up-regulated genes in adipo-, chondro- and osteo-induced WJ-MSCs as compared to the controls. *SLPI* encodes a serine protease inhibitor, a protein belonging to antimicrobial peptides involved in immune responses; however, Manna et al. [27] reported that *SLPI* and other antimicrobial peptides were expressed naturally in canine WJ-MSCs. In terms of osteogenic differentiation of MSCs, SLPI was demonstrated to improve the migration ability of BM-MSCs and to upregulate the expression of genes involved in osteogenic differentiation [28]. Similarly, Choi et al. [29] reported that SLPI increased the viability and promoted the differentiation and mineralization of osteoblasts on a titanium surface. The role of SLPI in chondrogenic and adipogenic differentiation of WJ-MSCs remains to be uncovered. However, SLPI was shown to be produced in the chondrocytes of human articular cartilage and in intervertebral disc fibrochondrocytes [30,31]. In addition, SLPI is expressed in adipocytes and in adipose tissue, where it participates in the resolution of inflammation [32,33]. Although the direct role of SLPI in the differentiation of WJ-MSCs has not been demonstrated yet, the scientific literature and the current results suggest that SLPI is involved in this process.

Similar to *SLPI*, *H19* (H19 Imprinted Maternally Expressed Transcript) was up-regulated in WJ-MSCs subjected to adipogenic, chondrogenic and osteogenic differentiation as compared to the controls. The product of this gene is a long non-coding RNA (lncRNA) acting as tumor suppressor; however, it has also been implicated in osteogenic differentiation of human MSCs. Liang et al. [34] revealed that H19 was up-regulated during osteogenesis in MSCs and accelerated osteoblast differentiation. Moreover, H19 was identified as one of the critical lncRNAs for osteogenic differentiation of human umbilical cord MSCs. Its overexpression resulted in increased ALP (Alkaline Phosphatase) activity and higher expression of the osteogenic markers [35]. Similarly, chondrogenic differentiation of human umbilical cord MSCs was promoted by the overexpression of H19 [36], while the adipogenic differentiation of BM-MSCs and adipose tissue-derived MSCs (ADSCs) was inhibited [37,38]. The current results reveal that H19 is involved in osteogenic, chondrogenic and adipogenic differentiation of WJ-MSCs.

Both *IL1RL1* (Interleukin 1 Receptor Like 1) and *PROK1* (Prokineticin 1) were up-regulated in adipo- and osteo-induced WJ-MSCs; however, none of them have been implicated in the differentiation of WJ-MSCs yet. *IL1RL1* encodes a receptor for IL-33 (Interleukin 33) and is involved in immune responses. Although this cytokine’s RNA was expressed in human osteocytes, osteoblasts, adipocytes and BM-MSCs, the *IL1RL1* was not expressed in these cells in basal conditions [39]. Schulze et al. [40], on the other hand, reported the expression of *Il1rl1* in murine primary osteoblasts during their differentiation. In addition, mRNA encoding IL1RL1 was found in preadipocytes, adipocytes and white adipose tissue [41]; therefore, it seems that the overexpression of *IL1RL1* in osteo- and adipo-induced WJ-MSCs confirms their differentiation. The role of PROK1 in osteogenic and adipogenic differentiation of MSCs has not yet been described; however, based on the current results, it is one of the factors involved in this process. PROK1 was shown to exert proangiogenic role and induce proliferation and differentiation in enteric neural crest cells in mice. Furthermore, PROK1 promotes proliferation of adrenal gland-derived endothelial cells and modulates the growth and survival of neurons and hematopoietic stem cells [42].

*DLK1* (Delta Like Non-Canonical Notch Ligand 1) constitutes the last of the top five overexpressed genes in adipo-induced WJ-MSCs. It encodes preadipocyte factor 1 (PREF1), which was already implicated in the adipogenic differentiation of MSCs as an early negative regulator of adipogenesis, although this effect seems to be cell-type specific [43]. Zhang et al. [44] revealed that some cultures of MSCs from human umbilical cord blood expressed PREF1. However, no correlation between PREF1 expression and ability of these cells to differentiate into adipocytes was observed. In the case of BM-MSCs and ADSCs, the adipogenic differentiation was accompanied by *DLK1* downregulation [45]. On the contrary, Morganstien et al. [46] reported an increase in the expression of *DLK1* during the first 7 days of adipogenic differentiation of fetal MSCs. The elevated expression of *DLK1* in the current study might suggest that WJ-MSCs acquired the features of preadipocytes.

The remaining gene among the top five up-regulated genes in WJ-MSCs subjected to osteogenic differentiation is *RORB* (RAR Related Orphan Receptor B). Similar to the previously mentioned gene, *RORB* was acting as a suppressor of murine osteoblast differentiation in vitro [47]. In addition, *RORB* was associated with the age-related bone loss; however, in vitro studies were conducted on mouse osteoblast model [48]. To date, no studies regarding the role of RORB in WJ-MSCs or even MSCs have been published, and the current results indicate that it is involved in osteogenic differentiation of WJ-MSCs. However, its exact mechanism of action is yet to be elucidated.

Beyond the aforementioned *SLPI* and *H19*, *CILP* (Cartilage Intermediate Layer Protein), *DPT* (Dermatopontin) and *OCA2* (OCA2 Melanosomal Transmembrane Protein) were overexpressed in WJ-MSCs subjected to chondrogenic differentiation. Both CILP and DPT constitute extracellular matrix proteins of human cartilage. CILP, synthesized by the chondrocytes, was localized in the middle zone of the articular cartilage [49]. In line with the current results, *DPT* was up-regulated in glucocorticoid-induced chondrogenic differentiation of human BM-MSCs [50]. While the overexpression of *CILP* and *DPT* seem to confirm the successful chondrogenic differentiation of WJ-MSCs, *OCA2* has not been associated with chondrogenesis yet, although it is involved in the transport of tyrosine and in melanocyte differentiation [51]. Moreover, Lecorguille et al. [52] revealed that maternal glycemic index changes in overweight women were related to the *OCA2* gene in cord blood. To date, this is the first report on the involvement of *OCA2* in chondrogenic differentiation of WJ-MSCs.

The genes that are the mostly overexpressed in neuro-induced WJ-MSCs are distinct from genes in osteo-, chondro- and adipo-induced WJ-MSCs. The top five genes up-regulated in WJ-MSCs subjected to neurogenic differentiation include *SPHKAP* (SPHK1 Interactor, AKAP Domain Containing), *DTX1* (Deltex E3 Ubiquitin Ligase 1), *MT1H* (Metallothionein 1H), *MT1F* (Metallothionein 1F) and *ZBTB16* (Zinc Finger and BTB Domain Containing 16), all of which were somehow implicated in neurogenesis or nervous cells’ function. SPHKAP plays a role in the modulation of sphingosine kinase 1 (SPHK1), and its overexpression resulted in reduced SPHK1 activity [53]. SPHK1 converts sphingosine into sphingosine-1-phosphate (S1P), a lipid concentrated in the brain, essential for proper brain function. Recently, SphK1/S1P axis was implicated in synaptic vesicle endocytosis [54]. The role of DTX1 in nervous system development was demonstrated mostly in animal models. Dtx1 was reported to promote rat and murine neural progenitor cell differentiation into oligodendrocytes [55]. In Zebrafish, *Dtx1* was primarily transcribed in the developing nervous system and induced neuronal and glial differentiation [56]. Metallothioneins are released by astrocytes act as intracellular metal binding molecules and participate in neuroprotection, homeostasis, transport and detoxification of metal ions. Metallothioneins are up-regulated in neurodegenerative disorders and in metabolic stress; both MT1H and MT1F were up-regulated in substantia nigra and frontal cortex of Parkinson’s disease patients [57]. Zbtb16, on the other hand, was reported to be involved in neuronal differentiation in Zebrafish [58] and exerted neuroprotective effect after nerve injury in mice [59]. Although it is clear that the top five up-regulated genes in neuro-induced WJ-MSCs are somehow associated with neural function, most of studies revealing this association were conducted in animal models. To date, our results demonstrate for the first time the role of these genes in neurogenic differentiation of human WJ-MSCs.

In summary, the current results indicate that both osteo- and adipo-induced WJ-MSCs share many similarities regarding the mostly overexpressed genes, while the neuro-induced WJ-MSCs are quite distinctive from the other three groups. Both *SLPI* and *H19* were up-regulated in WJ-MSCs subjected to osteogenic, chondrogenic and adipogenic differentiation, and the role of these genes in such differentiation has already been suggested. However, these results were obtained utilizing cells mainly from tissues other than Wharton’s jelly. Some of the remaining genes discussed in the current study have already been somehow implicated in osteogenic (*IL1RL1*, *RORB*), adipogenic (*DLK1*), chondrogenic (*DPT*) or neurogenic (*DTX1*, *ZBTB16*) differentiation, although in most cases these results were obtained from the experiments conducted on animal cells. *CILP* (up-regulated in chondro-induced WJ-MSCs) and *SPHKAP*, *MT1F* and *MT1H* (up-regulated in neuro-induced WJ-MSCs) were associated with the cartilage and nervous system function, respectively, although their role in the differentiation of MSCs remains unclear. *PROK1* (up-regulated in adipo- and osteo-induced WJ-MSCs) and *OCA2* (up-regulated in chondro-induced WJ-MSCs) have not yet been associated with this kind of differentiation or even with the related tissues, such as bone, adipose tissue or cartilage. Therefore, it seems that both of these genes constitute novel markers of WJ-MSCs differentiation.

In conclusion, the current results provide an insight into the transcriptomic changes occurring during the differentiation of WJ-MSCs and enable the identification of novel markers involved in this process, which might serve as a point of reference for future studies. Although further transcriptomic and proteomic studies are required to develop safe and efficient cellular therapies, the identification of differentially expressed genes involved in the process of in vitro differentiation enables the deeper understanding of the physiology of WJ-MSCs. The clinical utilization of WJ-MSCs in regenerative medicine on a more regular basis than currently conducted clinical trials still requires many years of research and proper optimization and standardization of the preparation of WJ-MSCs to ensure reliability and reproducibility. Nonetheless, the current results constitute a valuable molecular reference that sheds further light on the process of WJ-MSCs differentiation, and hopefully, this knowledge may serve as a starting point for further studies that can be applied in clinical practice. In addition, the differentiated WJ-MSCs, especially in the form of spheroids, might serve as a 3D tissue model for drug testing and development, especially when the cells of the required tissue are not easily accessible.

## 4. Materials and Methods

### 4.1. Material Collection

Twenty samples of umbilical cords were obtained during healthy full-term deliveries in the Gynecological Obstetric Clinical Hospital of Poznan University of Medical Sciences. The study was approved by the Ethics Committee of Poznan University of Medical Sciences (237/19 and 199/21) and performed according to the recommendations of the Declaration of Helsinki. Patients included in the study were 24–40 years old, and each mother provided the written consent for umbilical cord collection. Approximately 15 cm long samples of collected umbilical cords were placed in cold Dulbecco’s phosphate-buffered saline (DPBS; Merck, Darmstadt, Germany) with the addition of 10 U mL^−1^ penicillin, 10 mg mL^−1^ streptomycin and 25 µg mL^−1^ amphotericin B (Antibiotic Antimycotic Solution; Merck, Darmstadt, Germany) and processed within 24 h in the laboratory.

### 4.2. Isolation of Wharton’s Jelly-Derived Mesenchymal Stem Cells 

The processing started with double tissue washing to remove any blood remaining in the samples, using Dulbecco’s phosphate-buffered saline (DPBS; Merck, Darmstadt, Germany) with the addition of 10 U mL^−1^ penicillin, 10 mg mL^−1^ streptomycin and 25 µg mL^−1^ amphotericin B (Antibiotic Antimycotic Solution; Merck, Darmstadt, Germany). Subsequently, the umbilical cords were cut into 1 cm wide pieces, and the 2–3 mm Wharton’s jelly fragments were resected using forceps. After tissue mincing with a scalpel, the Wharton’s jelly was placed in 1 mg mL^−1^ collagenase type I (Gibco, Life Technologies, Waltham, MA, USA) for 24 h at 37 °C in a shaker. After incubation, the sample was centrifuged at 500× *g* for 20 min. Supernatant was removed from above the cell pellet, which was subsequently resuspended in DPBS and centrifuged at 500× *g* for 10 min. Following supernatant removal, the cell pellet was resuspended in 4 mL of culture medium consisting of Dulbecco’s Modified Eagle’s medium (DMEM, Merck, Darmstadt, Germany), with an addition of 10% fetal bovine serum (FBS, Merck, Darmstadt, Germany), 4 mM of L-glutamine (Merck, Darmstadt, Germany) and 10 U mL^−1^ penicillin, 10 mg mL^−1^ streptomycin and 25 µg mL^−1^ amphotericin B (Antibiotic Antimycotic Solution; Merck, Darmstadt, Germany).

### 4.3. In Vitro Cell Culture

The primary cell cultures were established using only samples with more than 85% viability, which was determined via the ADAM Automatic Cell Counter (NanoEntek, Waltham, MA, USA). The cells were maintained at 37 °C in a humified atmosphere of 5% CO_2_ in 25 cm^3^ culture flasks, with culture medium change every three days. After reaching 90% confluency, the cells were passaged using 0.25% trypsin solution (Merck, Darmstadt, Germany); the culturing was carried out up to the third passage. Daily observation of cellular morphology was performed via an inverted phase-contrast microscope (Olympus IX70, Olympus, Tokyo, Japan).

### 4.4. Flow Cytometry Analysis

Flow cytometry analysis was performed using half of the detached cells obtained during the third passage. Cells ready for evaluation using flow cytometry technique were subjected to immunophenotype analysis using direct fluorescence reaction. The expression of stem cell-specific antigens was determined using monoclonal antibodies, a list of which is provided in Table 1. Staining for differentiation markers such as CD44, CD90, CD105, CD31, CD73, CD45 and CD34 was carried out according to the following protocol. A 5 µL volume of antibodies for each of the identified antigens and 100 µL of the cell suspension were added to 5 mL reaction tubes. Samples were mixed and incubated for 15 min at room temperature without light. Next, 500 µL of buffer containing 1.5% paraformaldehyde was added to the samples to fix the binding between antigen and antibody. The whole mixture was incubated for 10 min, and then the samples were washed twice in phosphate-buffered saline (PBS) (Merck, Darmstadt, Germany) using centrifugation for 5 min at 1500 rpm at room temperature, removing the supernatants from the cell pellet each time. After a second centrifugation, cell pellets were finally resuspended in 150 µL of PBS, and the stained samples were then subjected to acquisition using a FACS Aria flow cytometer (Becton Dickinson, Franklin Lakes, NJ, USA). The data obtained using cytometric analysis were evaluated using FACS Diva software (Version 6.1.2) (Becton Dickinson, Franklin Lakes, NJ, USA).

### 4.5. Multilineage Differentiation

Cells were cultured until the third passage and then counted with the use of the ADAM Automatic Cell Counter (NanoEntek, Waltham, MA, USA). Subsequently, the appropriate amounts of cells were allocated for osteogenic, neurogenic, chondrogenic and adipogenic differentiation. After differentiation period, half of the cultures were stained to confirm differentiation and the other half was subjected to RNA isolation. Cells isolated from three umbilical cords were subjected to differentiation and subsequent RNA sequencing.

#### 4.5.1. Osteogenic Differentiation

Osteogenic differentiation began after the cells seeded on six-well culture plates at a concentration of 1 × 10^5^ cells per well reached 100% confluency. For this purpose, in half of the wells, the standard culture medium was changed to commercially available Mesenchymal Stem Cell Osteogenic Differentiation Medium (PromoCell, Heidelberg, Germany), while in the other half of the wells, the standard culture medium was used. Each plate contained cells isolated from separate umbilical cord. During the differentiation period, which lasted for 14 days, the medium was changed every 72 h. After the differentiation regimen, the cells were washed with PBS and fixed with Saccomanno Fixative solution (Morphisto GmbH, Offenbach am Main, Germany) for 30 min. For calcium deposit staining, Alizarin Red S (Sigma-Aldrich, Saint Louis, MO, USA) was applied for 15 min in darkness (as advised by the manufacturer’s protocol), and the results were observed with the use of an inverted phase-contrast microscope (Olympus IX70, Olympus, Tokyo, Japan).

#### 4.5.2. Neurogenic Differentiation

For neurogenic differentiation, 4 × 10^3^ cells/cm^2^ were seeded into each well of a six-well culture plate and cultured in the standard culture medium (changed every second day) until 60–80% confluency was reached. Subsequently, in half of the wells, the Mesenchymal Stem Cell Neurogenic Differentiation Medium (PromoCell, Heidelberg, Germany) was applied for seven days, while in the other half of the wells, the standard culture medium was used as a control. After seven days of differentiation, 0.5% cresyl violet staining for Nissl bodies was performed. For that purpose, the cell layer was washed with PBS and fixed with Saccomanno Fixative solution (Morphisto GmbH, Offenbach am Main, Germany) for 30 min at room temperature, and then washed again twice with PBS. The cell monolayer was stained with 0.5% cresyl violet, which was filtered using a 0.22 µm syringe filter (Millex, Merck, Germany). The staining lasted for 30 min at room temperature, and then the cell layer was washed three times with PBS. Finally, the results of staining were observed with the use of an inverted phase-contrast microscope (Olympus IX70, Olympus, Tokyo, Japan).

#### 4.5.3. Chondrogenic Differentiation

For chondrogenic differentiation, 3 × 10^5^ cells per well were seeded on Nunc 96-well Round Bottom Microwell Plate (Thermo Scientific, Waltham, MA, USA) to obtain spheroids. After 48 h, the spheroids were formed, and chondrogenic differentiation was conducted using the Mesenchymal Stem Cell Chondrogenic Differentiation Medium (PromoCell, Heidelberg, Germany) in half of the wells, while in the other half, the obtained spheroids served as controls and were cultured in the standard culture medium. Differentiation was carried out for 21 days, during which both media were changed every third day. To confirm differentiation and aggrecan deposition, Alcian Blue (Sigma-Aldrich, Saint Louis, MO, USA) staining was performed. For this purpose, the spheroids were fixed for 3 h at room temperature using Saccomanno Fixative solution (Morphisto GmbH, Offenbach am Main, Germany), after washing with PBS. Then, the Saccomanno Fixative solution was removed, and the spheroids were washed twice with distilled water. Alcian Blue, further used for staining, was previously filtered with the use of 0.22 µm syringe filter (Millex, Merck, Germany), and the staining procedure lasted for 45 min. Afterwards, a destaining solution was used to remove the dye, according to the manufacturer’s protocol. An inverted phase-contrast microscope (Olympus IX70, Olympus, Tokyo, Japan) was utilized to examine the results of staining.

#### 4.5.4. Adipogenic Differentiation

For adipogenic differentiation, 1 × 10^5^ cells per well were seeded in six-well culture plates and cultured until they reached 80–90% confluency. Subsequently, the standard culture medium was changed to the Mesenchymal Stem Cell Adipogenic Differentiation Medium (PromoCell, Heidelberg, Germany) in half of the wells. In the other half of the wells, cells were cultured in the standard culture medium to serve as controls. After 14 days of differentiation, with culture medium changed every third day, the cell monolayer was washed with PBS and fixed with Saccomanno Fixative solution for 30 min at room temperature. Subsequently, a distilled water was used to wash the cells followed with 5 min incubation with 60% isopropanol. Cell staining was performed for 3 min with Oil Red O (Sigma-Aldrich, Saint Louis, MO, USA), and then an inverted phase-contrast microscope (Olympus IX70, Olympus, Tokyo, Japan) was utilized to examine the results.

### 4.6. RNA Isolation

Both differentiated cells and controls were subjected to RNA isolation. The first step was the cell detachment with 0.25% trypsin solution. Subsequently, cells were suspended in 1 mL of TRIzol (Thermo-Fischer Scientific, Waltham, MA, USA) and immediately frozen at −80 °C. RNA was isolated using phase separation with chloroform, and isopropanol was used for RNA precipitation from the aqueous phase. Then RNeasy Mini kit was utilized for total RNA purification. RNA was eluted in 30 µL of RNAse/Dnase free water, subjected to quality assessment and stored at −80 °C. The Qubit™ RNA BR/HS Assay Kit (Thermo Fisher Scientific, Waltham, MA, USA) and the Agilent RNA 6000 Nano/Pico Chip (Agilent, Santa Clara, CA, USA) and the Bioanalyzer 2100 instrument (Agilent, Santa Clara, CA, USA) were used to RNA quantification and quality assessment. Both RIN values (6.9–10) and the concentration (6.2–335.0 ng/µL) fulfilled the criteria for library preparation.

### 4.7. RNA-seq

RNA samples with the input of 10 ng were used for library preparation with SMARter Stranded Total RNA-seq Pico Input Mammalian v3 Kit (Takara Bio, Kusatsu, Japan). After cDNA synthesis, the ribosomal RNA was depleted and the library was amplified in 15 PCR cycles. Libraries exhibited a quantity range of 32.1–64.4 nM, which was appropriate to pass the criteria for successful library preparation (more than 4 nM). Subsequently, the denaturation of libraries and dilution to final loading concentration (300 pM) were performed, and sequencing was conducted on NovaSeq 6000 S4 flowcell (Illumina, San Diego, CA, USA) with the aim to reach 60 M PE reads. For individual lane loading, the NovaSeq XP workflow was utilized and raw sequenced data were demultiplexed as well as QC metrics were generated. All quality control parameters were met by all of the samples but adapter content. Cutadapt (Available at: https://journal.embnet.org/index.php/embnetjournal/article/view/200, accessed on 28 May 2023) [60] was used to trim adapters and low-quality sequences. Then, the alignment of the trimmed raw reads to the human reference genome (hg19) from the Ensembl database was performed using STAR software (version 2.5.2b) [61]. featureCounts (Available at: https://academic.oup.com/bioinformatics/article/30/7/923/232889, accessed on 28 May 2023) [62] was used to obtain the overall summarization results, including the number of successfully assigned reads with unnormalized counts. Deseq2 library [63] was used for determination of differential expression.

### 4.8. Bioinformatical and Statistical Analysis

The analysis involved tabular data that included details about fold change, adjusted *p*-value, and normalized counts for each comparison. This data was analyzed using a BioConductor repository with the statistical programming language R (version 4.1.2; R Core Team 2021). To identify differentially expressed genes (DEGs), certain criteria were applied: an absolute fold change greater than two and a *p*-value with a false discovery rate (FDR) correction below 0.05. The outcomes of this selection were visualized through volcano plots, which depicted the total count of up-regulated and down-regulated genes. Next, to construct Venn diagrams, the “VennDiagram” package was utilized that enabled the visualization of the overlapping or shared elements between multiple sets or groups for a better ability to interpret the relationships and intersections among various gene sets or categories [64]. The DEGs obtained from each comparison were further analyzed using the DAVID bioinformatics tool, which stands for Database for Annotation, Visualization, and Integrated Discovery [65]. The gene symbols of the DEGs were uploaded to DAVID using the “RDAVIDWebService” BioConductor library [66]. Subsequently, the significantly enriched Gene Ontology (GO) terms from the GO BP Direct database were identified. To ensure the reliability of the results, the *p*-values of the selected GO terms were corrected using the Benjamini–Hochberg correction method [67].

Additionally, Gene Set Enrichment Analysis (GSEA) using the “cluster profiler” library was performed. The objective of this analysis was to determine the degree of depletion or enrichment in Gene Ontology (GO) terms using a normalized enrichment score (NES) and its corresponding *p*-value. A bar chart was then generated to summarize the most significant enrichment and depletion scores, highlighting ontology groups with the highest enrichment scores (highest NES values) and those with the most depleted scores (lowest NES values). Moreover, the enrichment plots for the top five enriched and depleted GO terms were created, providing a more detailed visualization of the enrichment levels. To explore the connections between the DEGs and the biological pathways or processes they are involved in, the “pathfinder” library for identification and visualization was employed [68]. “PathfindR” offers the advantage of detecting these relationships, which is particularly valuable in studying complex biological systems. A graph-based approach to visualize the relationships between the DEGs was employed, where the genes were represented as edges and the selected biological processes were depicted as central nodes that allowed us to explore the connections between gene expression levels and specific biological processes.

To identify functional protein partners among the input gene lists, Metascape was utilized [69]. Metascape serves as a comprehensive resource for analyzing and interpreting gene and protein function, pathway analysis and protein–protein interaction (PPI) network analysis. For the PPI network analysis, the minimum required interaction score was set to medium confidence (0.4). When the PPI network consisted of more than three nodes, the Detection (MCODE) algorithm was applied to uncover clusters that were directly associated with genes within the network [70]. Additionally, MCODE assigned a unique color to each cluster based on the *p*-value, providing further insights into the generated network.

## Figures and Tables

**Table 1:** The details of the antibodies used in the current study, including the type of the fluorochrome, isotype, clone and producer.

Antibody	Fluorochrome	Isotype	Clone	Producer
CD90	FITC	IgG1	REA897	Miltenyi Biotec (Bergisch Gladbach, Germany)
CD105	APC	IgG1	REA794	Miltenyi Biotec
CD73	PE	IgG1	REA804	Miltenyi Biotec
CD44	PE	IgG1k	DB105	Miltenyi Biotec
CD45	PerCP	IgG2ak	5B1	Miltenyi Biotec
CD34	PE	IgG2ak	AC136	Miltenyi Biotec
Isotype control IgG1k	PE	IgG1k	IS5-21F5	Miltenyi Biotec
Isotype control IgG1	FITC	IgG1k	IS5-21F5	Miltenyi Biotec
Isotype control IgG2a	PerCP	IgG2a	S43.10	Miltenyi Biotec
Isotype control IgG2a	PE	IgG2a	S43.10	Miltenyi Biotec
REA Control (S)	APC	IgG1	REA293	Miltenyi Biotec
REA Control (S)	PE	IgG1	REA293	Miltenyi Biotec

## Data Availability

All of the data discussed in this work, if not already included in the manuscript, are available from the corresponding author on reasonable request.
